# Novel targeted siRNA-loaded hybrid nanoparticles: preparation, characterization and in vitro evaluation

**DOI:** 10.1186/s12951-015-0124-2

**Published:** 2015-09-26

**Authors:** Nneka Dim, Maryna Perepelyuk, Olukayode Gomes, Chellappagounder Thangavel, Yi Liu, Robert Den, Ashakumary Lakshmikuttyamma, Sunday A. Shoyele

**Affiliations:** Department of Pharmaceutical Science, School of Pharmacy, Thomas Jefferson University, Philadelphia, PA USA; Department of Radiation Oncology, Thomas Jefferson University, Philadelphia, PA USA

**Keywords:** Nanoparticles, Targeted siRNA delivery, siRNA, Hybrid nanotechnology, Controlled release, Release kinetics

## Abstract

**Background:**

siRNAs have a high potential for silencing critical molecular pathways that are pathogenic. Nevertheless, their clinical application has been limited by a lack of effective and safe nanotechnology-based delivery system that allows a controlled and safe transfection to cytosol of targeted cells without the associated adverse effects. Our group recently reported a very effective and safe hybrid nanoparticle delivery system composing human IgG and poloxamer-188 for siRNA delivery to cancer cells. However, these nanoparticles need to be optimized in terms of particle size, loading capacity and encapsulation efficiency. In the present study, we explored the effects of certain production parameters on particle size, loading capacity and encapsulation efficiency. Further, to make these nanoparticles more specific in their delivery of siRNA, we conjugated anti-NTSR1-mAb to the surface of these nanoparticles to target NTSR1-overexpressing cancer cells. The mechanism of siRNA release from these antiNTSR1-mAb functionalized nanoparticles was also elucidated.

**Results:**

It was demonstrated that the concentration of human IgG in the starting nanoprecipitation medium and the rotation speed of the magnetic stirrer influenced the encapsulation efficiency, loading capacity and the size of the nanoparticles produced. We also successfully transformed these nanoparticles into actively targeted nanoparticles by functionalizing with anti-NTSR1-mAb to specifically target NTSR1-overexpressing cancer cells, hence able to avoid undesired accumulation in normal cells. The mechanism of siRNA release from these nanoparticles was elucidated to be by Fickian diffusion. Using flow cytometry and fluorescence microscopy, we were able to confirm the active involvement of NTSR1 in the uptake of these anti-NTSR1-mAb functionalized hybrid nanoparticles by lung adenocarcinoma cells.

**Conclusions:**

This hybrid nanoparticle delivery system can be used as a platform technology for intracellular delivery of siRNAs to NTSR1-overexpressing tumor cells.

## Background

siRNAs have a high potential to silence critical molecular pathways that are pathogenic. They can potentially restore sensitivity of different types of cancers to conventional/targeted therapies by silencing some pathways responsible for reducing or eliminating the sensitivities of tumors to known therapies [1]. The emergence of this novel technology has consequently led to multiple siRNA-based agents reaching various stages of clinical trials. The discovery of RNAi interference has simultaneously encouraged the development of various delivery systems to help to fully harness the benefits of siRNA in cancer therapy. This is more so, since the use of siRNAs in cancer therapy has been limited by a lack of efficient nanotechnology-based delivery system to ensure an efficient and safe delivery of this unique technology to specific cells while avoiding healthy cells in the process [2, 3]. Our group recently reported the use of a novel hybrid nanoparticle delivery system for the efficient and safe delivery of siRNA against mutant KRAS in A549 lung adenocarcinoma cells [4]. This nanoparticle technology takes advantage of the benefits derived from human immunoglobulin G (human IgG) and poloxamer-188 (polyoxyethylene-polyoxypropylene block co- polymer), for stable and efficient siRNA delivery [4]. Human IgG is the main component of these nanoparticles encapsulating the loaded siRNA. The inclusion of human IgG antibody helps to reduce/eliminate well documented immunogenic reaction experienced with most nanoparticle formulations [4]. Poloxamer-188, a nonionic triblock copolymer, has been previously used as a stealth polymer to prevent macrophageal uptake of nanoparticles hence circumventing the reticulo-endothelial system (RES) during systemic circulation. It was suggested that it reduces opsonization by serum proteins, hence reducing macrophage uptake [5, 6]. The delivery of anti-mutant KRAS-siRNA using these unique nanoparticles led to a stable knockdown of the target gene. Consequently, a restoration of sensitivity of A549 cells to EGFR-tyrosine kinase inhibitors was demonstrated [4]. The loaded siRNA was also protected from serum nuclease showing these nanoparticles are capable of delivering stable siRNAs to the cytoplasm of target cells [4]. Overall, these novel hybrid nanoparticles showed a great potential as a platform technology for safe and effective delivery of siRNA for oncogene knockdown in cancer cells. However, these nanoparticles still need to be optimized in terms of particle size, nanoparticle yield, encapsulation efficiency, and loading capacity. In this study, we investigate how certain nanoprecipitation parameters could be explored in order to optimize the size and loading efficiency of siRNA. Further, several reports have shown that active targeted drug delivery has allowed better delivery of loaded drugs (payload) to cancer cells [7–9]. In this present study, we explored the possibility of functionalizing these siRNA-loaded hybrid nanoparticles with anti-neurotensin receptor 1 monoclonal antibody (anti-NTSR1 mAb) in order to convert these nanoparticles to an active targeted nanoparticle delivery system for delivering siRNAs to non-small lung cancer cells (NSCLC) using NTSR1 as a delivery target. NTSR1 has been shown by immunohistochemistry to be overexpressed in approximately 60 % of lung adenocarcinoma [8]. In early stages of NSCLC, NTSR1 was one of the first 50 genes upregulated and associated with disease-free survival [10]. We hope to take advantage of this over expression in NSCLC to achieve an optimized targeting of siRNA to NSCLC by conjugating anti-NTSR1-mAb to the surface of our hybrid nanoparticles. The conjugation of anti-NTSR1 mAb to these nanoparticles was confirmed using FT-IR and fluorescence spectroscopy. Using anti-mutant KRAS-siRNA, and siGLO-green as our model siRNAs, we hoped to verify the involvement of NTSR1 in the internalization of siRNA-loaded hybrid nanoparticles in A549 and H23 lung adenocarcinoma cell lines. Further, we aimed to elucidate the mechanisms and kinetics of siRNA release from these nanoparticles in different physiological conditions using various mathematical models including zero-order, first order, Higushi, Hixson-Croswell and Korsmeyer-Peppas models [11, 12].

## Results

### Nanoparticle Preparation and characterization

**Fig. 1:**
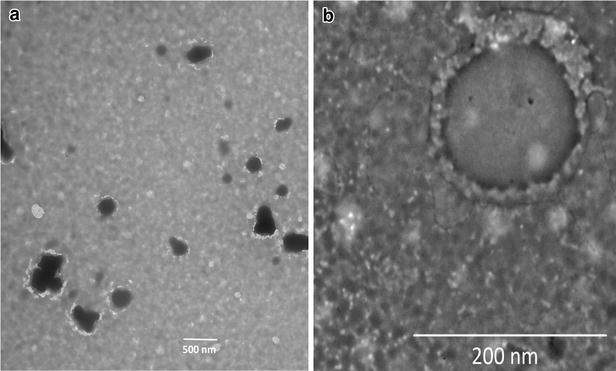
Diagrammatic representation and TEM micrograph of the produced nanoparticle. **a** Nanoparticles at lower magnification. **b** Nanoparticles at higher magnification to show the internal arrangement of the components

siRNA-loaded hybrid nanoparticles composing of human IgG in the inner layer and poloxamer-188 in the outer layer were prepared using our previously reported nanoprecipitation process [4]. The nanoparticles formed were mostly spherical in shape, as seen in Fig. 1a, irrespective of the conditions used. The multilayered structure of the nanoparticles was confirmed using transmission electron microscope as seen in Fig. 1b. The effects of human IgG concentration and magnetic stirring rate on particle size, PDI, zeta potential, encapsulation efficiency and loading capacity of hybrid nanoparticles are presented in Table 1. Table 1 shows that the concentration of human IgG and the magnetic stirring rate influenced the particle size, encapsulation efficiency (EE) and loading capacity (LC) of corresponding nanoparticle formulations. However, no such influence was observed for the PDI and zeta potential. EE and LC were calculated from the following equations:

$$ \% {\text{ EE}} = \left( {A - B} \right)/A \times 100 $$

$$ \% {\text{ LC}} = \left( {A - B} \right)/C \times 100 $$

where A is the total amount of siRNA, B is the free siRNA, and C is the weight of nanoparticles in grams.

**Table 1:** The effects of variable parametes on particle size, PDI, zeta potential, encapsulation efficiency and loading capacity of nanoparticles (Mean ± SD, n = 3)

Nanoparticle batch	Particle size (NM)	PDI	Zeta potential (mV)	Encapsulation efficiency (%)	Loading capacity (%)
IP-1	672.4 ± 17.9	0.08 ± 0.02	+17.1 ± 0.8	41.1 ± 0.2	0.71 ± 0.02
IP-2	424.6 ± 12.4	0.04 ± 0.01	+17.3 ± 0.4	50.2 ± 0.4	1.23 ± 0.03
IP-3	135.4 ± 5.4	0.07 ± 0.03	+16.7 ± 0.2	60 ± 0.4	2.04 ± 0.06
IP3-anti-NTSR1-mAb	140.2 ± 2.4	0.2 ± 0.04	0.0 ± 0.3	N/A	N/A
IP-4	716.1 ± 15.2	0.1 ± 0.04	+17.4 ± 0.4	35.4 ± 0.4	0.62 ± 0.04
IP-5	545.3 ± 14.3	0.06 ± 0.03	+17.9 ± 0.3	40.3 ± 0.3	0.81 ± 0.03
IP-6	389.2 ± 11.4	0.06 ± 0.05	+17.5 ± 0.4	48.5 ± 0.5	1.21 ± 0.05
IP-7	820.5 ± 12.4	0.08 ± 0.03	+17.4 ± 0.5	30.3 ± 0.1	0.53 ± 0.02
IP-8	799.3 ± 11.4	0.1 ± 0.04	+16.9 ± 0.6	37.3 ± 0.3	0.65 ± 0.03
IP-9	589.3 ± 10.4	0.09 ± 0.05	+17.2 ± 0.3	42.6 ± 0.4	0.82 ± 0.04

*PDI* polydispersity index

The conjugation of anti-NTSR1-mAb to the surface of IP-3 nanoparticle formulation led to an increase in the particle size of these functionalized nanoparticles as compared to the corresponding non-functionalized nanoparticles. A change in zeta potential was also observed with the functionalized nanoparticles producing a zeta potential of 0.0 while the corresponding non-functionalized nanoparticles produced a zeta potential of +16.7. All the batches produced nanoparticles with narrow particle size distribution as indicated by the PDI values.

### Conjugation of anti-NTSR1-mAb to hybrid nanoparticles

**Fig. 2:**
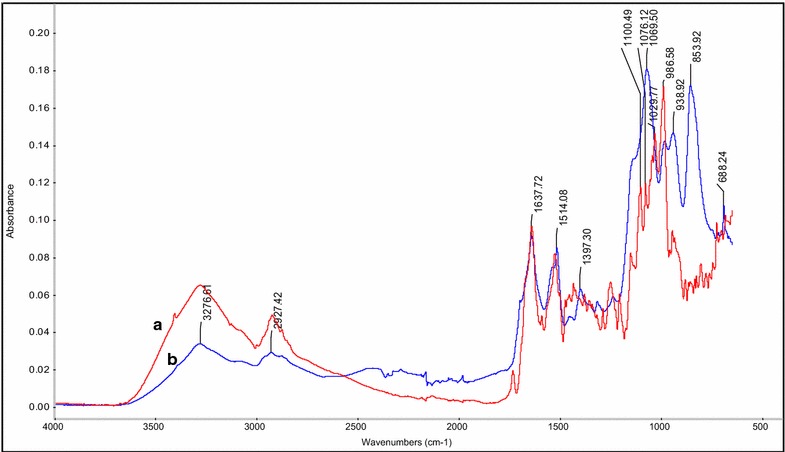
-FI-IR spectra showing the conjugation of anti-NTSR1-mAb to hybrid nanoparticles (*a*) incomparison to non-functionalized nanoparticles (*b*)

80 % of the thiolated anti-NTSR1-mAb used in the conjugation reaction was found to couple to the nanoparticles from the protein analysis performed using Total Protein Kit. Further, a total of 20 mg of anti-NTSR1-mAb was calculated to be attached to 1 g of functionalized hybrid nanoparticles. FT-IR was used to confirm the covalent conjugation of anti-NTSR1-mAb to the nanoparticles. Figure 2 demonstrates the distinctive differences between the spectra generated for the functionalized and non-functionalized hybrid nanoparticles. To verify the presence of anti-NTSR1-mAb on the surface of the nanoparticles, the fluorescent intensity obtained from the coupling of FITC-labelled sheep antimurine IgG to anti-NTSR1-mAb on the surface of the functionalized nanoparticles was compared to that of the non-functionlized nanoparticle control. Table 2 demonstrates the increased fluorescent intensity of the anti-NTSR1-mAb nanoparticles when compared to the other samples suggesting the presence of ant-NTSR1-mAb on the surface of the hybrid nanoparticles.

**Table 2:** Fluorecent intensities of anti-NTSR1-mAb functionalized hybrid nanoparticles was compared to that of the non-functionalized, PBS solution and free sheep antimurine IgG labelled with FITC (Mean ± SD, n = 3)

Sample	Fluorescent intensity
Anti-NTSR1-mAb functionalized nanoparticles	2435.1 ± 7.5
Non-functionalized nanoparticles	436.2 ± 3.4
PBS	314.3 ± 5.1
Sheep antimurine IgG-FITC	1134 ± 10.3

### In vitro release study

**Fig. 3:**
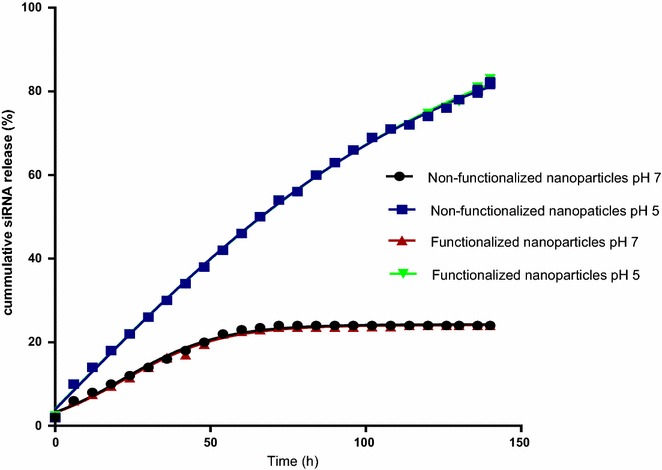
Comparison of in vitro siRNA release profile from siRNA encapsulated anti-NTSR1-mAb functionalized and non-functionalized nanoparticles at pH 5 and 7. siRNA was more efficiently released at pH 5 due to the superior solubility of human IgG at that pH. Functionalization of the nanoparticles did not seem to affect the release rate at each of the pH values. n = 3 for each sample point

The release of anti-mutant KRAS siRNA from both anti-NTSR1-mAb functionalized hybrid nanoparticles and non-functionalized hybrid nanoparticles was compared at pH values 5 and 7.4. Figure 3 demonstrates the lack of any discernable differences in the release profile of siRNA from both nanoparticle formulations (functionalized and non-functionalized) at the respective pH values. However, a limitation in the release of siRNA from both nanoparticle formulations was demonstrated at pH 7.4 when compared to the release profile at pH 5.

### Release kinetics and mechanisms of siRNA release

Using several mathematical models including zero-order, first-order, Higuchi, Hixson–Crowell, and Korsmeyer–Peppas models, the kinetics and mechanisms of siRNA release from both functionalized and non-functionalized hybrid nanoparticles were evaluated using correlation values (R^2^) and release parameters determined from the results of model fitting of the release profiles. The results presented in Tables 3 and 4 suggest that the release of the siRNA from both types of nanoparticles at pH 5 was by Fickian diffusion. A definite mechanism could not be elucidated for the release at pH 7.4.

**Table 3:** Mathematical models and parameters based on siRNA release data from non-functionalized nanoparticles

PH	Correlation (R)					n value for Korsmeyer–Peppas
	Zero order	First-order	Higuchi	Hixson–Crowell	Korsmeyer–Peppas	
7.4	0.6578	0.3456	0.6877	0.3455	0.5456	0.35
5	0.4567	0.5673	0.9856	0.4563	0.9856	0.67

**Table 4:** Mathematical models and parameters based on siRNA release data from NTSR1-functionalized nanoparticles

PH	Correlation (R)					n value for Korsmeyer–Peppas
	Zero order	First-order	Higuchi	Hixson–Crowell	Korsmeyer–Peppas	
7.4	0.4537	0.6784	0.4579	0.4536	0.4563	0.34
5	0.4567	0.4675	0.9754	0.5647	0.9789	0.64

### NTSR1 expression in non-small lung cancer cells

**Fig. 4:**
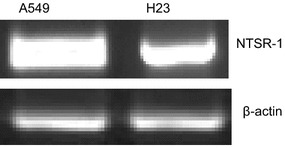
Reverse transcriptase PCR showing the expression of neurotensin receptor 1 (NTSR1) in A549 and H23 cell lines

Reverse transcriptase PCR was used to confirm the expression of NTSR1 in two non-small cell lung cancer cells: A549 and H23 to ensure that these cells actually express the receptor. Figure 4 demonstrates the positive expression of this target receptor in both cells.

### Cellular uptake of anti-NTSR1-mAb functionalized hybrid nanoparticles

**Fig. 5:**
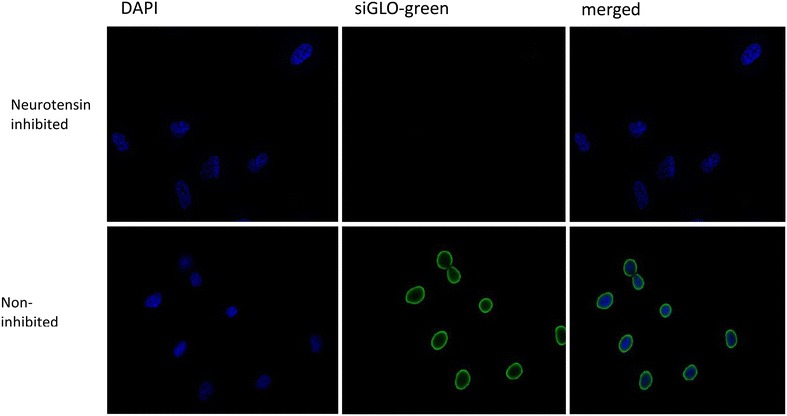
Fluorescence micrograph showing the delivery of siGLO into the cytosol of A549 cells using the NTSR1-mAb-functionalized hybrid nanoparticles. The upper panel shows the inhibition of siGLO delivery following an initial treatment of the cells with neurotensin

**Fig. 6:**
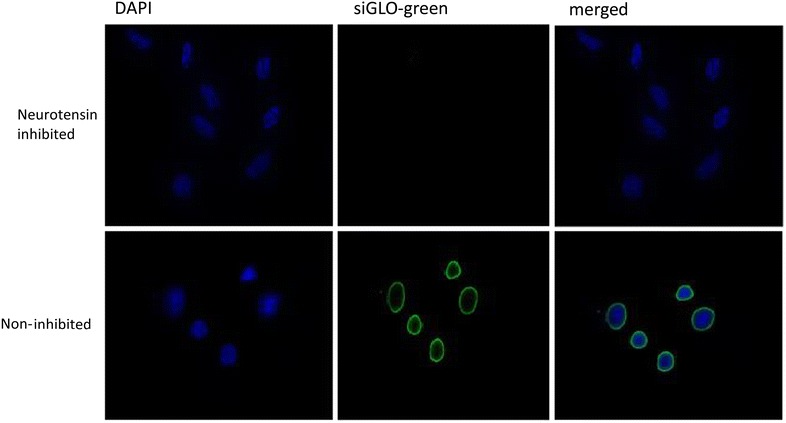
Fluorescence micrograph showing the delivery of siGLO into the cytosol of H23 cells using the NTSR1-mAb-functionalized hybrid nanoparticles. The *upper panel* shows the inhibition of siGLO delivery following an initial treatment of the cells with neurotensin

The involvement of neurotensin receptor 1 in the internalization of siGLO-loaded NTSR1-functionalized hybrid nanoparticles by A549 and H23 cells was accessed using both fluorescence microscopy and flow cytometry analysis. Figures 5 and 6 demonstrate the inhibitory effect of excess concentration of neurotensin on the internalization of siGLO as demonstrated by the absence of siGLO in the cytosol of the respective cell lines after 6 h. However, the absence of neurotensin in the cell culture enhanced the internalization of siGLO in the cells as demonstrated Figs. 5 and 6.

**Fig. 7:**
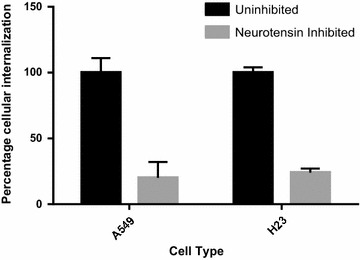
Probing the effect of inhibition of neurotensin receptor 1 (NTSR1) with neurotensin on the internalization of siRNA-loaded targeted hybrid nanoparticles in A549 and H23 cells using flow cytometry (n = 3)

We were able to quantify the effect of neurotensin receptor 1 on the internalization of siGLO using flow cytometry. Figure 7 demonstrates a significant inhibition of the internalization of siGLO since only approximately 20 % internalization was observed in both cells following inhibition by neurotensin.

## Discussion

The main objective of this study was to optimize critical parameters in our recently reported novel hybrid nanoparticles composing human IgG and poloxamer-188. This is to make them more efficient as a nanotechnology-based delivery platform for siRNAs. We also aimed to transform these hybrid nanoparticles into an active targeted platform for delivery of siRNAs to NTSR1 expressing tumors by covalently attaching anti-NTSR1-mAb to the surface of these nanoparticles and to confirm the involvement of NTSR1 in the uptake of these nanoparticles by cancer cells. The mechanism of release of encapsulated siRNA in different physiological pH conditions was also elucidated.

**Fig. 8:**
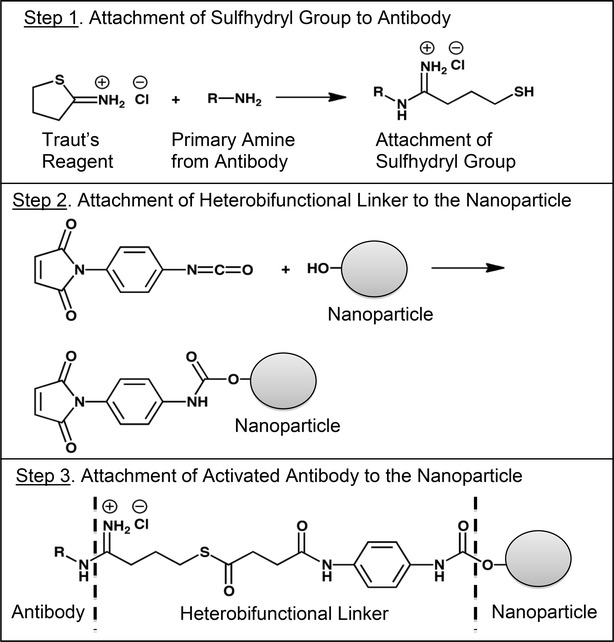
Reaction schemes for the attachment of anti-NTSR1-mAb to hybrid nanoparticles

The impact of particle size on the cellular internalization efficiency of nanoparticles has been variously reported [15–17]. It has also been previously reported that the size of the nanoparticles plays a key role in their adhesion to and interaction with biological cells [18]. In view of this, it is extremely important for any nanoparticle technology intended for intracellular delivery of siRNAs to be able to produce size-tuneable nanoparticles. In this study, we explored the effect of certain factors such as the concentration of the human IgG and the magnetic stirring rate during the nanoprecipitation process on critical parameters such as particle size, siRNA encapsulation efficiency (EE) and loading capacity (LC). Data in Table 2 demonstrates that an increase in the concentration of human IgG in the nanoprecipitation medium led to a decrease in the EE and LC. This result is consistent with previously reported chitosan nanoparticles produced by increasing the concentration of chitosan in the production process [11, 12]. This is probably due to the fact that an increase in the amount of human IgG led to an increase in the number of nanoparticles formed which subsequently led to lesser amount of siRNA available for encapsulation in each nanoparticle since the concentration of the siRNA was kept consistent for all the nanoparticle batches produced. In case of the size of nanoparticle produced, an increase in the magnetic stirring rate led to a decrease in the size of the nanoparticles produced. A decrease in nanoparticle size was achieved due to an increase in the shear rate brought about by the increase in the stirring rate of the magnet during the production of the nanoparticles [19]. The particle size of the nanoparticles were stable following storage at ambient and 4 °C conditions for 1 month. Following the optimization of the particle size, EE and LC, the nanoparticle batch with the optimum parameters in terms of particle size, EE and LC was selected as the nanoparticle formulation for further development. Nanoparticle formulation IP-3 with particle size 135 nm, EE of 60 % and LC of 2.04 % was selected for functionalization with anti-NTSR1-mAb to transform it to an actively targeted hybrid nanoparticle system for siRNA delivery. To achieve the conjugation of anti-NTSR1-mAb to prepared hybrid nanoparticles, the primary amines of anti-NTSR1-mAb was thiolated using 2-iminothiolane (Traut’s reagent) as previously reported [3, 11]. PMPI was used as a sulfhydryl and hydroxyl-reactive linker and was conjugated to the hydroxyl groups present in poloxamer-188 on the surface of the hybrid nanoparticles [13]. The isocyanate end of PMPI reacted with the hydroxyl group on the nanoparticles to form carbamate linkage while the maleimide end reacted with the sulfhydryl groups in the thiolated anti-NTSR1 [13]. The reaction steps are shown in Fig. 8. The particle size of anti-NTSR1-mAb functionalized nanoparticles was higher than the corresponding non-functionalized nanoparticles. The increase in size may be attributed to the conjugation of the mAb to the surface of the nanoparticles. Further, the fact that several centrifugation steps and lyophilisation were involved in the conjugation process may also contribute to the increase in particle size [11, 20]

FT-IR (Fig. 2) was used to confirm the conjugation of anti-NTSR1-mAb to the nanoparticles. The spectrum of non-functionalized hybrid nanoparticles has an absorption peak at 3276 cm^−1^. This peak has been attributed to the stretching vibration from the OH hydroxyl group in poloxamer-188 present on the surface of the nanoparticles [21]. This peak can also be seen in the spectrum for anti-NTSR1-mAb functionalized nanoparticles, however, with lesser intensity. This decrease in intensity suggests the conjugation of the hydroxyl group to another molecule. The linker PMPI used for the conjugation of anti-NTSR1-mAb to the nanoparticles reacts with the hydroxyl groups in poloxamer-188 as shown in the reaction in Fig. 8. The absorption peak at 853 cm^−1^ in the anti-NTSR1-mAb functionalized nanoparticles has been attributed to the para-substituted aromatic ring in PMPI. This peak is conspicuously absent in the non-functionalized nanoparticles spectrum suggesting the presence of the linker on the surface of the functionalized nanoparticles. The absorption peak at 1149 cm^−1^ in both spectral confirms the presence of the C–O–C stretching vibration from the ether group in poloxamer-188. The absorption peak at 688 cm^−1^ seen in the anti-NTSR1-mAb functionalized nanoparticles spectrum is due to the thioether bond (C–S–C) connecting the anti-NTSR1-mAb to PMPI on the surface of the nanoparticles [22]. This important peak is conspicuously absent in the spectrum of the non-functionalized nanoparticles. The differences in the both spectra confirm the successful conjugation of anti-NTSR1-mAb to the hybrid nanoparticles.

Fluorescence spectroscopy was also used to confirm the presence of anti-NTSR1-mAb on the surface of the nanoparticles [9]. Nanoparticles functionalized with antibody demonstrated a significantly higher fluorescent intensity because of the presence of anti-NTSR1-mAb on the surface of the nanoparticles. This allowed the nanoparticles to bind significantly to sheep antimurine IgG-FITC when compared to the non-functionalized nanoparticles.

Recently, active targeted nanoparticles have enabled better and more efficient delivery of payload to cancer cells. These actively targeted nanoparticles are usually achieved via covalent conjugation of a specific mAb to nanoparticle surface, which recognizes and bind to a specific receptor/biomarker expressed in tumors [7, 9, 23]. However, it has recently been reported that the transformation of these nanoparticles to actively targeted nanoparticle by surface functionalization with mAb could inadvertently inhibit the release of the payload in physiological fluids [11]. To ensure that the anti-NTSR1-mAb functionalization of these novel hybrid does not inhibit the release of the loaded siRNA in certain physiological fluids, we attempted to elucidate the mechanisms and release kinetics of siRNA from both functionalized and non-functionalized hybrid nanoparticles at pH 7 and 5. These pH values were chosen in order to simulate the pH of different body fluids including that of a cancer microenvironment. Figure 3 demonstrates the pH sensitive nature of these nanoparticle delivery systems. At pH value of 7, very limited amount of the loaded antimutant-KRAS siRNA (approximately 20 %) was released throughout the study period. The limited release of siRNA at pH 7 could be attributed to the reduced/limited solubility of IgG at the pH. Proteins are known to have limited solubility at pH values close to their isoelectric point (pI) [24, 25]. Since the pI of IgG is 7, its solubility at neutral pH values is quite limited. This makes it difficult for encapsulated siRNAs to be released at this pH value, hence possibly limiting their release extracellularly. However, at slightly acidic pH value of 5, an optimal siRNA release of approximately 100 % was obtained due to the solubility of IgG at this pH. An optimal release of the loaded siRNA is very desirable at pH 5, as pH 5 represents the acidic condition of the endosome/lysosome [26, 27]. This is important since endosome is the normal site of intracellular siRNA release [4, 28]. Further, the lack of differences in the release profile of the non-functionalized and anti-NTSR1-mAb-functionalized nanoparticles at the respective pH values suggests that the presence of the anti-NTSR1-mAb on the surface of the functionalized nanoparticles does not affect the release of the loaded siRNA. This is contrary to a previous report which showed that conjugated mAb on the surface of nanoparticles adversely affected the release of the payload, erlotinib [11]. The reason for this difference could be attributed to the fact that antimutant-KRAS siRNA has a high aqueous solubility, allowing the uninhibited diffusion of siRNA through the hybrid nanoparticles despite the presence of mAb on the surface. On the contrary, erlotinib has a very poor aqueous solubility; hence, the presence of mAb inadvertently inhibited its diffusion through chitosan nanoparticles.

In order to elucidate the mechanisms and kinetics of the release of siRNA from the hybrid nanoparticles, mathematical models were applied.

From Tables 3 and 4, it is very clear that the release of antimutant-KRAS siRNA from the functionalized and non-functionalized hybrid nanoparticles at pH 5 fitted well to both Higuchi and Korsmeyer–Peppas release kinetics. The correlation factors for both groups of nanoparticles were well above 0.95.

The Higuchi model describes the release of payload from a system as a square root of the time dependent processed based on Fickian diffusion [11, 29]. This model is defined by Eq. 1.

1 $$ Q_{t} = Q_{0} e + K_{H} t^{1/2} $$

where, *Q*_*0*_ is the initial amount of payload, *Q*_*t*_ is the cumulative amount of payload at time t, K_H_ is the Higuchi constant.

The Korsmeyer-Peppas model describes release of payload from a polymeric system. For this model, pure diffusion represents the controlling release mechanism when *n* ≥ 0.5 [11, 30]. This model is defined by Eq. 2.

2 $$ Q_{t} = K_{KP} t^{n} $$

where *K*_*KP*_ is the Korsmeyer-Peppas constant and *n* is the release exponent describing the release mechanism [30].

The fact that the release of siRNA from both functionalized and non-functionalized hybrid nanoparticles fitted well with both Higuchi and Korsmeyer–Peppas further confirms that the release of siRNA from the anti-NTSR1-mAb functionalized is not inhibited by the presence of the anti-NTSR1-mAb on the surface of the nanoparticles at this pH. Further, it also confirms that the release of siRNA from these nanoparticles was by Fickian diffusion. This is in agreement with previuos reports showing that the release of payloads from most nanoparticles were by Fickian diffusion [12]. However, the release of siRNA from both functionalized and non-functionalized hybrid nanoparticles at pH 7 did not fit any of the models studied. This could mean that the release of siRNA from these hybrid nanoparticles at this pH involves a combination of mechanisms or that the mechanism of release involved was not covered in this study.

Following the successful conjugation of anti-NTSR1-mAb to the surface of the hybrid nanoparticles, it was essential to confirm the involvement of NTSR1 in the uptake of these nanoparticles by lung adenocarcinoma cells known to overexpress this receptor. Previous report by Takahashi et al. [31] had shown that both A549 and H23 lung adenocarcinoma cells overexpress this receptor. Reverse transcriptase PCR in Fig. 4 confirms the expression of NTSR1 in these cells. The role of this receptor in the uptake of siGLO-loaded anti-NTSR1-mAb functionalized hybrid nanoparticle was confirmed using both fluorescence microscopy and flow cytometer. An excessive amount of the neurotensin, the main ligand for this receptor was initially added to the culture media 60 min prior to the addition of the nanoparticles so as to occupy all available receptors [32]. Control experiments omitting the addition of neurotensin in the culture media for both cells were also carried out for comparison. Fluorescence microscopy (Figs. 5, 6) and flow cytometry (Fig. 7) confirm the specificity of the uptake of these nanoparticles in both A549 and H23 cells. The presence of neurotensin in excess amount inhibited the interaction of the anti-NTSR1-mAb functionalized hybrid nanoparticles. This is due to the fact that most of the known effects of neurotensin are mediated through its specific interaction with NTSR1 [33, 34]. NTSR1 has been reported to bind neurotensin at its extracellular surface [35], hence inhibiting the interaction of the anti-NTSR1-mAb functionalized hybrid nanoparticles with NTSR1 present on the surface of both A549 and H23 cells.

## Conclusions

We reported the production of tunable novel hybrid nanoparticles suitable for the intracellular delivery of siRNAs. The particle size, loading capacity and encapsulation efficiency of anti-mutantKRAS siRNA in these nanoparticles were optimized by exploring the influence of the concentration of human IgG in the nanoprecipitation medium and the rotation speed of the magnetic stirrer on critical parameters such as particle size, loading and encapsulation efficiency. These hybrid nanoparticles were successfully transformed into actively targeted nanoparticles by covalently binding anti-NTSR1-mAb to the surface. The involvement of the target receptor (NTSR1) in the uptake of these nanoparticles by lung adenocarcinoma cells was confirmed using both fluorescent microscopy and flow cytometry. The mechanism of siRNA release from these actively targeted hybrid nanoparticles was elucidated to be by Fickian diffusion. Altogether, this hybrid nanoparticle delivery system can be used as a platform technology for intracellular delivery of siRNAs to NTSR1-overexpressing tumor cells.

## Materials and methods

### Materials

Human IgG was purchased from Equitech Bio (Kerrville, TX, USA). Poloxamer-188, RNase-free water, 4,6-diamidino-2-phenylindole (DAPI), and fetal bovine albumin (FBS) were obtained from Fisher Scientific. Antineutotensin receptor 1-monoclonal antiboby (anti-NTSR1-mAb was purchased from Santa Cruz Biotechnologies. siRNA against mutated KRAS G12S was designed andpurchased from GE Dharmacon. siG12S sense and antisense sequences are GUUGGAGCUAGUGGCGUAGdTdT and CUACGCCACUAGCUCCAACdTdT respectively. siGLO-Green (6-FAM-labeled) was obtained from GE Dharmacon. N-[p-maleimidophenyl] isocyanate (PMPI), and 2-iminothiolane-HCl were obtained form Thermo Scientific. Neurotensin was purchased from Abcam (Cambridge, MA, USA)

### Cell line and cell culture

Adernocarcinoma cell lines A549 and H23, expressing KRAS mutation at G12S and G12C were obtained from American Type Culture Collection (ATCC), Rockville, MD. A549 cells were maintained in F12 K medium supplemented with 10 % FBS and 1 % antibiotics. H23 cells cells were maintained in RPMI supplemented with 10 % FBS and 1 % antibiotics. Both cells were kept in a humidified air atmosphere with 5 % carbon dioxide.

### Methods

#### siRNA-loaded nanoparticle preparation

Hybrid nanoparticles were prepared based on our previously reported protocol [4]. 50, 75 and 100 mg milligrams of excipient-free human IgG was dissolved in 0.01 N HCl containing 20 mg of poloxamer-188 and 187 μg of siRNA to make a 10 mL total solution in a 50 mL beaker. The final concentrations of human IgG in each solution amounted to 5, 7.5 and 10 mg/mL. These solutions were then slowly titrated with 0.01 N NaOH to bring the pH of the mixture to 7, which is the isoelectric point (pI) of human IgG as determined in our laboratory using isoelectric focusing. The nanoparticles were continuously mixed at different revolutions per minute (125, 250 and 350 rpm) on a magnetic stirrer for additional 10 min. The length of the magnetic stirrer was kept consistent at 13 mm. At the pI, siRNA-loaded nanoparticles were spontaneously precipitated. The colloidal suspension was then centrifuged with a micro-centrifuge (Eppendorf centrifuge 5418) at 2000 rpm for 5 min. The supernatant was decanted, and the nanoparticles were rinsed thrice with double distilled deionized water. Nano particles were then redispersed in water before being snap-frozen using liquid nitrogen. This was then loaded into a freeze- dryer (Labconco FreezeZone 4.6), and lyophilization was performed for 48 h. The different parameters used for each nanoparticle formulation is presented in Table 5.

**Table 5:** Variable parameters used in the formulation of different nanoparticle batches

Nanoparticle batch	Concentration of human IgG (mg/mL)	Concentration of poloxamer-188 (%w/v)	Magnetic stirring rate (rpm)	Amount of siRNA (µg)
IP-1	5	0.2	125	187
IP-2	5	0.2	250	187
IP-3	5	0.2	350	187
IP-4	7.5	0.2	125	187
IP-5	7.5	0.2	250	187
IP-6	7.5	0.2	350	187
IP-7	10	0.2	125	187
IP-8	10	0.2	250	187
IP-9	10	0.2	350	187

#### Thiolation of anti-NTSR1-mAb

Thiolation of anti-NTSR1-mAb was performed based on a method adopted from a previously used method [9]. Briefly, 1.3 × 10^−4^ M of Traut’s reagent (2-Iminothiolane.HCl) was prepared in phosphate buffered saline (pH 7.4). 500 µl of this solution was then added to 1 mL of 1.3 × 10^−6^ M (0.2 mg/mL) of anti-NTSR1-mAb solution. The reaction was stirred for 2 h at 25 °C. The mixture then centrifuged at 4000 rpm and 10 °C for 15 min using 30 kDa cutoff centrifugal ultrafilters (Millipore Corp) to exclude unreacted Traut’s reagent.

#### Activation of hybrid nanoparticles with heterobifunctional cross-linker

5 mg/mL of hybrid nanoparticles were dispersed in 0.1 M phosphate buffer (pH 7.2). This was then added to 2 mg/mL of N-[p-maleimidophenyl] isocyanate (PMPI), a heterobifunctional crosslinker that links sulfhydryl to hydroxyl groups [13]. PMPI was dissolved in 50 mM phosphate buffer (pH 8).The reaction was performed for 3 h at 25 °C, after which the activated nanoparticles were centrifuged at 4000 rpm at 10 °C for 15 min using 30 kDa ultrafilters (Millipore Corp.)

#### Preparation of anti-NTSR1-mAb functionalized hybrid nanoparticles

The activated nanoparticles were finally functionalized with the thiolated anti-NTSR1-mAb by adding 500 µL of 2 mg/mL thiolated anti-NTSR1-mAb to 4 mL of activated nanoparticle suspension (5 mg/mL) and then incubated for 3 h at 25 °C. The functionalized nanoparticles were then centrifuged for 30 min at 4000 rpm and 10 °C. unconjugated anti-NTSR1-mAb in the supernatant was quantified using Total Protein Kit (Micro Lowry, Sigma) based on the supplier’s instructions.

#### Nanoparticle characterization

Particle size distribution of the nanoparticles was measured by photon correlation spectroscopy (PCS) using ZetaSizer Nano ZS with DTS software (Malvern Instruments, UK). Pellets formed after centrifugation and rinsing were redispersed in deionized water using a pipet. Intensity autocorrelation was measured at a scattering angle (θ) of 173°. The Z-average and polydispersity index (PDI) were recorded in triplicate. For zeta potential measurement, samples were taken in a universal dip cell (Malvern Instruments) and the zeta potential was recorded in triplicate.

The morphology and internal arrangements of the components of the nanoparticles were characterized by transmission electron microscopy (TEM). A drop of nano-particle suspension was made onto a copper grid coated with carbon membrane and then air-dried. The nanoparticles were observed using a FEI Tecnai 12 TEM. Electron micrographs were captured with an AMT XR111 11 megapixel CCD camera.

#### Reverse Transcriptase PCR

Total RNA was isolated using Qiagen RNAeasy kit (Qiagen, Valencia, CA, USA), and reverse transcribed using Verzo cDNA kit (Thermoscientific, Waltham, MA, USA). PCR was carried out using 25 μl reaction mixtures containing 1.0  μl of cDNA, 1 × Qiagen buffer, 0.2 mM of dNTP mixture, 0.2 μM of each primer and 1.5 U of HotStar *Taq* (Qiagen, Valencia, CA, USA). Reactions consisted of 1 cycle at 95  °C for 15 min followed by 35 amplification cycles (94  °C for 1 min, 55  °C for 1 min and 72  °C for 1 min) for neurotensin receptor 1 (NR1). β-actin PCR was performed using the same conditions except for the annealing temperature (59 °C). Primer sequences of NR1,Forward-5′-CGTGGAGCTGTACAACTTCA-3′, reverse 5′-CAGCCAGCAGACCACAAAGG-3′ and β-actin, Forward 5′-CCAAGGCCAACCGCGAGAAGAT-3′, reverse 5′-TTGCTCGAAGTCCAGGGCGA-3′.

#### siRNA release study

Release of siRNA from non-functionalized and anti-NTSR1-mAb functionalized hybrid nanoparticles was investigated using pH 5 and 7.4 acetate buffer and PBS respectively. Nanoparticles were suspended in 0.5 mL of a buffered solution in a tubular cellulose dialysis membrane secured tightly at both ends. This was then incubated in 5 mL buffered solution reservoirs at 37 °C while the reservoir was gently agitated. The amount of siRNA released at different time points was analyzed and quantified for percentage cumulative release using ion-pair HPLC as detailed below.

#### Ion-pair HPLC

siRNA quantitative analysis was performed with a Waters 2695 separation module combined with a Waters 2998 photodiode array detector Alliance HPLC system (Waters, Milford, MA, USA). A Waters XSELECT HSS C18 column XP (4.6 × 150 mm) was used. 1 μL of siRNA sample was injected using 20 mM triethylamine–acetic acid (pH 7) and 5–12 % acetonitrile, gradient elusion as mobile phase. Analysis was performed at a flow rate of 0.2 mL/min. UV detection was performed at 269 nm, and chromatograms were recorded using Empower Pro software.

#### Fourier transform infrared spectroscopy (FTIR)

Spectra from non-functionalized hybrid nanoparticles and anti-NTSR1-mAb-functionalized hybrid nanoparticles were collected using a single-reflection attenuated total reflectance (ATR) with a diamond internal reflection crystal installed in a iS10 FTIR spectrometer (Thermo Fisher Scientific, Madison, WI, USA). Lyophilized powders were placed on the surface of the ATR crystal after background spectra had been collected. Spectra were collected after 64 scans at 4 cm^−1^ resolution. Data were analyzed using OMNIC software.

### Flourescence spectroscopy

To verify the presence of anti-NTSR1-mAb on the surface of the hybrid nanoparticles, fluorescein isothiocyanate (FITC)-labeled sheep antimouse immunoglobulin (IgG) was incubated with the functionalized and non-functionalized nanoparticles at 25 °C for 2 h at a ratio of 1:1000 [14]. The mixtures were then centrifuged at 13,000 rpm for 15 min. The pellets were washed twice with PBS in order to remove unattached FITC-labeled sheep antimouse IgG. Fluorescien labeled nanoparticles were then suspended in PBS. The fluorescence intensity of fluorescien dye was measured at λ ex 494 nm and λ em 525 nm using a fluorescent microplate reader. This was then compared to that of non-functionalized hybrid nanoparticles treated with FITC-labeled sheep antimouse IgG, PBS and FITC-labeled antimouse IgG.

#### Fluorescence microscopy

A549 and H23 cells (2 × 104 cells/well) were seeded in 8 well coated glass slides (Discovery Labware, USA) and incubated for 48 h. PBS washed cells were incubated with anti-NTSR1-functionalized siGLO-FAM (green)-loaded hybrid nanoparticles suspended in the RPMI medium (100 μg/mL) for 6 h. Corresponding cells were initially treated with 1000 molar excess of neurotensin, 60 min before being treated with the NTSR1-functionalized hybrid nanoparticles. Cells were washed with siGLO-FAM (green)-loaded hybrid nanoparticles also for 6 h. Cells were then washed with PBS, fixed with 2 % paraformaldehyde, and incubated at room temperature for 20 min. PBS washed cells were then blocked with 5 % BSA for 30 min at room temperature. Cells were stained with 4′,6-diamidino-2-phenylindole (DAPI) to visualize nucleus. Cells were then mounted and observed under a Leica DMI 6000B fluorescence microscope (Leica Microsystems, Exton, PA, USA).

### Flow cytometry

A549 and H23 cells were used to investigate the uptake of functionalized siGLO-FAM loaded hybrid nanoparticle. About 1 million cells/well were seeded in a 12 well plate and incubated for 48 h. Cells were then treated with 100 μg/mL the FITC conjugated nanoparticles resuspended in RPMI medium and incubated for 6 h. Corresponding cells were initially treated with 1000 molar excess of neurotensin, 60 min before being treated with the NTSR1-functionalized nanoparticles. Cells were washed with siGLO-FAM (green)-loaded hybrid nanoparticles also for 6 h. The cells were trypsinized and centrifuged at 1000 rpm for 5 min, and the pellet was washed and resuspended in PBS. The samples were then filtered through 0.75 µm cell strainer before being analyzed by flow cytometry (BDFACS caliber). 10000 cells were measured in each sample.

### Statistical analysis

Results are expressed as mean ± standard deviation (SD), unless otherwise indicated. The difference between two groups was determined by two-tailed Student’s t test. A p value of 0.05 was taken as statistically significant.
